# Perinatal Predictors and Mediators of Attachment Patterns in Preschool Children: Exploration of Children’s Contributions in Interactions with Mothers

**DOI:** 10.3390/children11081022

**Published:** 2024-08-21

**Authors:** Stefan Kurbatfinski, Jennifer Woo, Henry Ntanda, Gerald Giesbrecht, Nicole Letourneau

**Affiliations:** 1 Department of Community Health Sciences, Cumming School of Medicine, University of Calgary, Calgary, AB T2N 1N4, Canada; stefan.kurbatfinski@ucalgary.ca (S.K.); ggiesbre@ucalgary.ca (G.G.); 2 Owerko Centre, Alberta Children’s Hospital Research Institute, Calgary, AB T2N 1N4, Canada; henry.ntanda@ucalgary.ca; 3 Department of Psychiatry, University of Saskatchewan, Regina, SK S7N 0W8, Canada; jlw517@usask.ca; 4 Departments of Pediatrics, Cumming School of Medicine, University of Calgary, Calgary, AB T2N 1N4, Canada; 5 Owerko Centre, Faculty of Nursing and Cumming School of Medicine, Departments of Pediatrics, Psychiatry, and Community Health Sciences, University of Calgary, Calgary, AB T2N 1N4, Canada

**Keywords:** attachment, child health, child interactive behaviour, cortisol, stress, mediation, predictors

## Abstract

Insecure and disorganized attachment patterns in children are linked to poor health outcomes over the lifespan. Attachment patterns may be predicted by variables that influence the quality of children’s interactions with their primary caregivers/parents (usually mothers) such as prenatal and postnatal exposures and the children’s own behaviours in interactions. The purposes of this exploratory study were to examine: (1) prenatal predictors of children’s attachment patterns, and (2) postnatal mediators and moderators of associations between prenatal predictors and children’s attachment patterns, with adjustment for relevant covariates. Mother–child dyads (*n* = 214) from the longitudinal Alberta Pregnancy Outcomes and Nutrition (APrON) cohort were studied using valid and reliable measures. Hayes’ mediation analysis was employed to determine direct and indirect effects. Mothers’ prenatal cortisol levels directly predicted disorganized (versus organized) child attachment in unadjusted models. Children’s passivity (in adjusted models) and compulsivity (in unadjusted and adjusted models) in parent-child interactions mediated the pathway between mothers’ prenatal cortisol levels and children’s disorganized attachment patterns. Serial mediation analyses revealed that mothers’ cortisol levels predicted their children’s cortisol levels, which predicted children’s compulsivity, and, ultimately, disorganized attachment in both unadjusted and adjusted models. No predictors were correlated with children’s insecure (versus secure) attachment. This exploratory research suggests that prenatal exposure to mothers’ cortisol levels and children’s behavioural contributions to parent–child interaction quality should be considered in the genesis of children’s attachment patterns, especially disorganization. Interventions focused on parent-child interactions could also focus on addressing children’s behavioral contributions.

## 1. Introduction

Children’s attachment patterns are linked to long-term health outcomes [1,2,3] and thought to reflect the historical and concurrent quality of children’s interactions with their primary caregivers/parents, usually mothers [4,5]. The sensitivity and responsiveness of mothers to their growing infants during interactions have been theorized to explain later attachment patterns observed in children; however, meta-analytic evidence suggests weak associations, particularly for secure attachment [6,7,8]. This is concerning as many interventions focused on promoting secure attachment target maternal sensitivity and responsiveness in interactions, neglecting many other factors that could influence outcomes [9]. The association between mothers’ sensitivity or responsiveness with children’s attachment security may be confounded by factors such as exposure to perinatal depression or social support [10,11]. Both prenatal perceived stress [12] and physiologic stress, indexed by hypothalamic–pituitary–adrenal (HPA) axis cortisol activity [13], have been linked to mothers’ perinatal depression, which could in turn, influence children’s attachment patterns [14]. Children’s stress likely plays a role in the quality of children’s behaviours in parent–child interactions [15], something that is rarely studied [16,17,18]. Overall, children’s attachment patterns may be predicted by variables that influence the quality of their interactions with parents, including their own behaviours in interactions and their prenatal and postnatal exposure to depression and stress, as measured by cortisol. Exploratory analyses are needed to investigate the relevance and timing of these variables in the etiology of children’s attachment pattern development.

### 1.1. Children’s Attachment Patterns and Outcomes

Secure attachment is an organized pattern of parent–child relational behaviour that predicts children’s positive mental health, emotional regulation, peer relationship quality, and adaptability over the lifespan [2,3,4,19]. Insecure attachment is another organized pattern of attachment that is conversely associated with worsened child health across the lifespan such as difficulty with self-regulation [2,8,20]. Insecure organized attachment has two observable anxious subtypes: (1) avoidant, which is characterized by behaviours in which children demonstrate excessive self-reliance and avoid seeking help from parents, and (2) ambivalent/resistant, which is characterized by behaviours in which children demonstrate preoccupation with maintaining closeness to their parents [21]. A third pattern of insecure attachment is referred to as disorganized, characterized by both extremely avoidant and anxiously attached behaviours whereby children are unable to regulate their distress [22,23].

Secure attachment is thought to emerge from a child’s expectation, based on previous experiences, that their actions and emotions will be met with sensitive, responsive, and comforting actions from their parents [4]. In contrast, insecure attachment is thought to arise from experiences with a parent who rejects or ignores their child’s emotions and/or responds inconsistently to their child’s needs [21,24]. Disorganized attachment is thought to emerge from experiences with a parent who demonstrates frightening or atypical behaviours, such as dissociation, emotional withdrawal, or abusiveness in interactions with their child [18]. These parenting behaviours undermine children’s ability to organize adaptive responses, as the parent is simultaneously a source of comfort and fear [18,22,23]. While disorganized attachment is considered the most impairing attachment pattern [22,23], all three insecure patterns are linked to poor child health outcomes (e.g., internalizing and externalizing behavioural problems) [25,26], less optimal familial well-being, and disrupted dyadic interactions [18,27].

While systematic and meta-analytic reviews have shown that sensitive and responsive maternal behaviours during interactions with their children predict the security of children’s attachment, these findings explain less variance in attachment pattern development than theorized [6,8,9]. For secure attachment, the association with maternal sensitivity was *r* = 0.24 [6]. For disorganized attachment, the associations with maternal insensitivity, frightening or frightened maternal behaviour, maternal unresolved loss, maternal depression, and child maltreatment, were all deemed insufficient to explain the pattern [28,29,30]. Observed associations between parents’ behaviours in interactions and children’s attachment pattern are modest at best [6,7], suggesting other factors are important to consider.

### 1.2. Depression, Social Support, Cortisol, and Children’s Attachment Patterns

Globally, conservative estimates suggest approximately 15% of expectant and new mothers report symptoms consistent with perinatal depression [31,32,33,34,35,36], increasing the risk of emotional, behavioural, cognitive, physical, and neurodevelopmental challenges in children [37,38,39,40,41]. A systematic review revealed associations between more severe prenatal (but not postnatal) symptoms of maternal depression and disorganized attachment in children up to 24 months of age [42]. Prenatal depression has been linked to greater levels of the stress hormone cortisol in mothers during pregnancy [40,43,44] and to later disorganized attachment in 12-month-old children [14], but studies have also shown that depression is not associated with cortisol secretion among chronically depressed individuals [45]. Social support from intimate (e.g., marital) partners during pregnancy may buffer the negative impacts of mothers’ depression on their daily cortisol levels [46], indicating the potential role of mothers’ perceived social support as a moderator of the effects of depression and cortisol on children’s attachment patterns. The fetal programming hypothesis suggests that in utero conditions can alter and hardwire fetal development in anticipation of similar conditions when born, with potentially lifelong impacts on health and development [47]. Prenatal stressors, such as expectant mothers’ depression, have been associated with structural and physiological changes in the fetus, leading to behavioural and phenotypic changes that are related to neuropsychiatric disorders [48]. Exposure to high cortisol during pregnancy has been shown to alter fetal neurodevelopment [48], with impacts on children’s cognitive ability, behaviour, and emotional regulation [40,49,50]. Elevated prenatal cortisol in mothers has also been linked to increased cortisol reactivity in infants at 6 and 12 months of age [51], and cortisol responses have been observed to be misattuned among disorganized mother–infant dyads [52]. Moreover, disorganized infants demonstrate higher salivary cortisol levels compared to securely and insecurely organized attached children [53,54], revealing the importance of considering mothers’ and infants’ cortisol in the etiology of children’s attachment patterns. Whether social support interacts with depression and/or mothers’ and children’s cortisol in the pathway to attachment patterns has not been extensively studied, suggesting a need for further exploration.

### 1.3. Parent–Child Interaction and Child Attachment: Children’s Contributions

Interactions are bidirectional in nature, and, as infants age, they become increasingly influential contributors to the quality of parent–child interactions, termed ‘child effects’ [55,56,57]. More specifically, children’s behaviours may contribute to observable parent responses based on cues during interactions [55]. Cooperative infants are moderately aroused, attentive, and responsive, initiating behaviours and appearing comfortable in interactions with their parents [58]. Difficult infants are more aroused and display intense negative affect to elicit attention and cue their parents to their need to change their state [58]. Those deemed more passive are characterized as less engaged in interactions, becoming inattentive, appearing bored, and responding to little that happens around them [17]. Conversely, those with more compulsive tendencies may demonstrate precocious self-control, inhibiting behaviours deemed disagreeable to parents, complying readily with parents’ demands, and substituting negative displays of their emotions in favour of false positive ones [16]. All but cooperative infant behaviours challenge parents’ abilities to appropriately respond to, and meaningfully engage with, their infants [16,58]. These behaviours have been rarely studied in the context of parent–infant interactions linked to attachment [16,18].

The quality of parent–child interaction is undermined when mothers are depressed, as symptoms of low mood and fatigue contribute to less sensitivity and responsiveness to children’s needs [8,9,59,60,61]. Mothers’ prenatal depression has been associated with disorganized (versus organized) attachment in infants at 12 months of age, but only when mothers were observed to be less warm and positive in interactions at three months postpartum [14]; infants’ contributions to interaction quality were not considered in this study. Prenatal stress has been linked to infants’ negative behaviours (e.g., unpredictability) in interactions [62] that could, in turn, influence attachment pattern. Social support from intimate (e.g., marital) partners may buffer the negative impacts of maternal depression on mothers’ interactions with their children [15,63], but it is unknown whether social support affects infants’ interactive behaviours in the context of the development of attachment patterns. To our knowledge, whether perinatal depression, prenatal cortisol, or social support impacts infants’/children’s behaviours in their interactions with parents, in the pathway to attachment patterns has not been well studied.

### 1.4. Sociodemographic Factors 

Sociodemographic factors such as age, ethnicity, parity, education, or child sex may contribute to children’s attachment patterns. Mothers who are younger [64], low parity, primigravidae and/or inexperienced [64,65], and have low incomes [8] are more likely to have children with insecure or disorganized attachment patterns. Non-white mothers often lack equitable access to healthcare [66,67], which may limit their ability to access support in providing optimal care to their children and potentially increase the risk of insecure or disorganized child attachment [68]. Since cortisol levels tend to increase as pregnancy progresses [69,70], gestational age is valuable to consider when examining prenatal cortisol levels at different trimesters/time points. In predicting attachment patterns, adjustment for these variables is warranted. 

### 1.5. Rationale and Purpose of This Study

**Figure 1:**
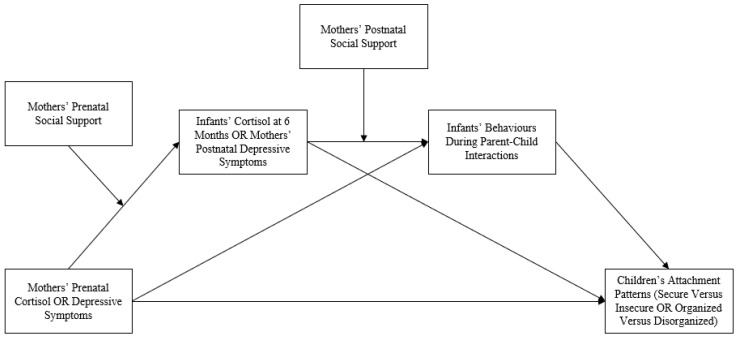
Model of the planned analysis.

While the contributions of parents in interactions with children have been well studied [6,7,9], both the predictors and outcomes of children’s contributions require attention and may offer additional insights into the genesis of attachment patterns. Though hypercortisolism has been linked to depression in adults, studies have also reported no change in baseline cortisol levels among chronically depressed individuals, and, at times, blunted cortisol responses [45], suggesting that other factors can contribute to depression. Investigating depressive symptoms and mothers’ prenatal cortisol levels as separate predictors of children’s attachment is therefore warranted. Since mothers’ depressive symptoms and prenatal cortisol levels have been associated with infants’ cortisol levels, infants’ behaviours during parent–child interactions, and mothers’ postnatal depressive symptoms, these variables were identified as important candidate mediators of the association between mothers’ prenatal cortisol and/or depressive symptoms and children’s attachment patterns, while also considering how social support may buffer the associated negative impacts of depressive symptoms and cortisol levels (Figure 1).

For these reasons, the purposes of this exploratory study were to investigate: (1) prenatal predictors of children’s attachment patterns, and (2) postnatal mediators and moderators of associations between prenatal predictors and children’s attachment patterns, with adjustment for relevant covariates. We hypothesized that more prenatal depressive symptoms and greater levels of prenatal cortisol in mothers would correlate with insecure (versus secure) and disorganized (versus organized) attachment in their children. Further, we hypothesized that infants’ cortisol levels at 6 months, mothers’ postpartum depressive symptoms, and children’s behaviours in parent–child interactions would mediate these associations, such that greater levels of infant cortisol and postnatal maternal depressive symptoms and less optimal infant behaviour during interactions with parents would positively mediate insecure or disorganized attachment patterns. Lastly, we hypothesized that social support would buffer the associations between prenatal and postnatal mediators and outcomes. To our knowledge, this exploration may be the first to investigate the roles of mothers’ prenatal stress (indexed by cortisol), perinatal depression, and perinatal social support, as well as children’s stress (cortisol) and contributions to parent–child interactions, in the pathway to attachment patterns. The findings offer potential in revealing novel targets for intervention to promote secure attachment and prevent disorganized attachment.

## 2. Methods

### 2.1. Study Design, Setting, and Sample

This study utilized data from the longitudinal, prospective Alberta Pregnancy Outcomes and Nutrition (APrON) cohort, specifically the Fetal Programming (FetalPro) sub-study [71]. APrON has been following mothers and their children for over 15 years, collecting data on maternal and child mental and physical health. The FetalPro subsample was designed to collect additional data such as mothers’ and children’s stress (i.e., cortisol), parent–child interaction quality, and children’s attachment patterns [71]. The inclusion criteria for APrON were that women had to be ≥16 years, early in pregnancy (<27 weeks), able to complete questionnaires in English, and living in central or southern regions of Alberta, Canada without plans to move. Mothers who planned to leave the study region during pregnancy up to 3 months postpartum were excluded. For the FetalPro sub-study, families from APrON who volunteered to support the additional data collection objectives of FetalPro were included. More information is published elsewhere [71]. A total of 214 mother–child dyads constituted the initial sample for this data analysis. All mothers were self-reported primary caregivers of their children. 

Alberta is a western province situated in Canada with a relatively younger demographic age group, higher median income level than other North American regions, and high educational attainment prevalence [72]. Just over half of the Albertan population identify as a visible minority, suggesting that the province is relatively diverse in culture [72]. Lastly, Alberta’s healthcare system is publicly funded and provided to citizens universally for necessary and primary care appointments at no additional cost. Relative to the general population of Alberta, APrON mothers were typically older, were more often married, reported higher income levels, and attained higher educational levels, but they were similar in terms of birth outside of Canada [71].

### 2.2. Procedures, Data Collection, and Measures Used

Mothers were mailed questionnaires for completion and biological and attachment data were collected in the laboratory at various time points. The study team was composed of researchers from various academic and research backgrounds and only certified, trained members conducted specialized assessments; the procedures used have been published and detailed elsewhere [71,73,74]. Mothers provided sociodemographic data assessed at the first study visit in the first or second trimester of pregnancy on family income (dichotomous measure of less than CAD 70,000 per year or greater than or equal to CAD 70,000 per year), education (dichotomous measure of less than university degree or university degree or more), and mothers’ age (continuous measure in years). Gestational age of the child at birth was collected at the 3-month postpartum study visit (continuous measure of number of weeks).

### 2.3. Prenatal Predictors

Mothers’ Depressive Symptoms: The Edinburgh Postnatal Depression Scale (EPDS) is a 10-item self-reported questionnaire that assesses depressive symptoms [75]. It was administered in early (<21 weeks’ gestation) and late pregnancy (≥21 weeks’ gestation); details may be found in other publications [73,76]. Higher scores are indicative of more depressive symptoms and scores above 12 are generally consistent with a physician diagnosis of major depressive disorder. The EPDS demonstrates high psychometric quality and is widely used for measuring depressive symptoms [77,78]. A meta-analysis of 53 studies confirmed moderate-to-high sensitivity (0.81) and specificity (0.88) using a cut-off score of 11 in screening for major depression among pregnant and postpartum women [79]. The internal consistency reliability of the EPDS in this study was high with Cronbach’s alpha of 0.82.

Mothers’ Cortisol: Prior to 21 weeks’ gestation, diurnal suites of salivary cortisol were collected over two days from mothers. Samples were used to calculate the area under the curve ground (AUCg) cortisol estimates, adjusted for waking times, as is considered best practice [80,81]. We also calculated the area under the curve increase (AUCi) cortisol estimates from waking and 30 min post-waking cortisol data points, reflecting the increase in cortisol in the morning [82,83]. As associated with chronic stress, two days of diurnal salivary cortisol measurements were aggregated to produce more stable estimates than that attainable with one day of measurement [82,83]. For more details on saliva collection and analysis methods, please see previous publications [15,82,83].

Mothers’ Social Support: The Social Support Effectiveness Questionnaire (SSEQ) is a self-report, 35-item, 5-point Likert measure that assesses the emotional, informational, task, and negative social support experienced within the past 3 months [84]. It was administered in early (<21 weeks’ gestation) and late pregnancy (≥21 weeks’ gestation). Higher scores indicate more perceived social support. The SSEQ demonstrates high reliability and validity among perinatal women, with internal consistency reliability with Cronbach’s alpha values greater than 0.80 and strong factor structure [84,85]. The internal consistency reliability of the SSEQ in this study was high with Cronbach’s alpha of 0.89.

### 2.4. Postnatal Mediators

Mothers’ Depressive Symptoms: The EPDS was used [75] (see prenatal maternal depressive symptoms). Postnatally, maternal depressive symptoms were measured at 3 months.

Mothers’ Social Support: The SSEQ was used [84] (see prenatal social support). Postnatally, maternal social support was measured at 3 months.

Infants’ Cortisol: Suites of infant salivary cortisol were collected before and after administration of the Laboratory Temperament Assessment Battery [86], a proxy for an acute psychosocial stressor, at 6 months postpartum. AUC estimates quantified the infants’ cortisol levels with two different measures: (1) AUCi, to assess infant cortisol reactivity within 20 min of stress exposure, and (2) AUCg, to assess total cortisol secreted. For more details on these saliva collection and analysis methods used, please see previous publications [15,82,83].

Parent–Child Interaction Quality: The Child Adult Relationship Experimental (CARE)-Index was used to measure the quality of infants’ behaviours during interactions with their parents [58]. At 6 months of infant age, a 5-minute observational procedure was carried out, involving videotaping the mother and child playing freely with age-appropriate toys. In this study, infants’ contributions in interactions with their mothers, including cooperativeness, difficultness, passiveness, and compulsivity were the focus [58]. Total scores range from 0 to 14, with higher scores indicating more of the children’s behaviour. It is well validated [16,87,88,89,90,91] with a high degree of stability over time, producing interrater reliability values between *r* = 0.73 and 0.95 [90,92]. Author Dr. Nicole Letourneau is a reliable CARE-Index coder who supervised the administration of the measurements. Video recordings were coded by trained, blinded designates including an internationally regarded CARE-Index trainer at the Family Relations Institute, Florida, United States of America, achieving a 94.4% interrater agreement on the dominant observable constructs. Disagreements between coders were resolved by additional viewings and discussion of any construct. The details of these data collection methods used are published elsewhere [93].

### 2.5. Outcomes

Child Attachment: Ainsworth’s [21] and Main and Solomon’s [23] gold-standard coding schemes were applied to the Strange Situation Procedure (SSP) to measure child attachment at a mean age of 22 months. The SSP is a laboratory-based procedure, whereby a mother undertakes repeated separations and reunions with her child, sometimes in the presence of a stranger unknown to the child [94]. The SSP has sound psychometric properties with interrater reliability from *r* = 0.75 to 0.96 for the patterns of attachment [21,95] and patterns have been associated with developmental outcomes [24]. All observations were coded by a certified coder in ABC and D classifications trained by the laboratory of Alan Sroufe (Institute of Child Development, University of Minnesota, United States of America) and Marinus Van Ijzendoorn (University of Cambridge), respectively. All SSP videotapes were coded for patterns of attachment using standard categories of secure (Type B) and insecure with the subtypes of avoidant (Type A), ambivalent/resistant (Type C), and disorganized (Type D) [21,23]. For analyses, variables were created for Secure (Type B) versus Insecure (Types B, C, and D) and Disorganized (Type D) versus Organized (Type A, B, and C). A random 15% of the recordings were also re-coded by an independent expert coder, an internationally regarded trainer at the Sroufe laboratory. The Cohen’s kappa for interrater reliability was 0.73. 

## 3. Statistical Analysis

The statistical analyses employed were conducted on Statistical Analysis Systems (SAS) version 9.4 for Windows. Descriptive statistics (e.g., mean and standard deviation) characterized continuous variables. Frequencies and percentages were presented for categorical and ordinal variables. The data were evaluated for normality, linearity, homoscedasticity, independence, and multicollinearity as appropriate before running analyses. From the full set of possible variables, model building was based on the following activities. Bivariate Spearman correlations and Chi-square statistics were used to identify at least moderate associations between the attachment outcomes and the prenatal predictors or the postnatal mediators, allowing identification of variables for inclusion in models based on *p*-values less than 0.25. This threshold is suitable for exploratory analyses as more traditional levels such as 0.05 can fail to identify variables known to be important (Supplementary Materials, Table S1) [96]. To examine mediation of the included variables, serial multiple mediation analysis using Hayes PROCESS SAS macro version 4.3 was employed [97]. Missing data were handled using listwise deletion as they constituted less than 5% of the overall sample.

The objective of the models was to investigate the total and direct effects (a1, a2, b1, b2, d21, and c′), as shown by the standardized regression coefficients among the dependent and independent variables. Further, the indirect effects (a = a1*b1; b = a2*b2; c1 = a1*d21*b2) displayed changes in child attachment with the mothers’ prenatal cortisol levels mediated through postnatal variables (e.g., the children’s role in parent–child interactions and the children’s cortisol levels postnatally). A resampling procedure of 20,000 was used to calculate 95% confidence intervals (CIs). Indirect effects were significant when the 95% CI did not contain zero. All models were fitted using a random seed for reproducibility. With respect to power estimation, we relied on mediation expert Hayes’ approach of bootstrapping to maximize power to detect effects. According to Hayes, “an indirect effect is formed as a product of two effects with no agreed upon way of quantifying the magnitude of those effects or their product (something you need to do to assess the power to detect an effect of a given size)” [97], (p. 141). This application of bootstrapping resampling methods that approximated all theoretically possible sample distributions of the indirect effect is understood to generate a higher power than tests that apply a product of coefficient approach [97], (pp. 95-98). 

Covariates were chosen based on bivariate association linear regression analysis with attachment patterns. In the context of secure versus insecure attachment patterns, linear regression analysis revealed gestational age at birth and birthweight as moderately significant covariates (*p* < 0.25; Supplementary Materials, Table S2). For disorganized versus organized attachment patterns, ethnicity (non-white versus white individuals), household income (less than versus more than CAD 70,000), and birthweight were identified as moderately significant covariates (*p* < 0.25; Supplementary Materials, Table S2). Gestational age at the time of maternal cortisol data collection and early-pregnancy maternal depressive symptoms and social support were included as control variables in the models despite a lack of statistical association (Supplementary Materials, Table S1) since they are theoretically important variables in the etiology of children’s attachment patterns. For example, though depressive symptoms can be a proxy for experiences of stress [45], cortisol levels can vary based on the type and severity of depression [45], and self-reported depressive symptoms may not be accurately reported due to associated stigma [98]. By controlling for depressive symptoms, this reduced extraneous effects that derive from the adverse effects of mothers’ withdrawal, fatigue, and lack of motivation that are often reported through depressive symptoms [99] on children’s attachment patterns. Controlling for mothers’ perceived social support, which has been reported as pivotal to healthy perinatal mother–infant outcomes [46], was essential to consider the potential extraneous effects on children’s attachment patterns. 

For the first section of results, an unadjusted analysis followed by an adjusted analysis (including ethnicity, household income, birthweight, early-pregnancy depressive symptoms, and early-pregnancy social support as covariates) was undertaken. Since gestational age is highly correlated with mothers’ prenatal cortisol levels and cortisol was collected over a range of weeks in early pregnancy [69,70], mothers’ prenatal cortisol levels (AUCg) were residualized by running a linear regression where prenatal maternal cortisol was predicted by gestational age at the time of cortisol collection. Then, residuals were extracted from this analysis and employed as the predictor variable in the second set of mediation analyses. By using residuals, this ensured: (1) the effect of mothers’ prenatal cortisol was not confounded by gestational age at each trimester, (2) the effect of mothers’ prenatal cortisol levels was isolated, and (3) indirect effects reflected the mediator of interest [100,101].

## 4. Ethical Approval

Informed written consent was acquired from all participants prior to any data collection. This study received ethics approval from the University of Calgary Conjoint Health Research Ethics Board (ID# REB14-1702 for the APrON Study and ID# REB15-1200 for the FetalPro Substudy) and the University of Alberta Health Research Ethics Biomedical Panel (ID# Pro00002954).

## 5. Results

### Participant Demographics

After removal of the missing data based on variables used in the analyses, the analytical sample size for this study was 214 in unadjusted (based on available data for predictor and mediator variables) and 204 in adjusted analyses (based on available data for relevant covariates). More than three quarters of the mothers had, at minimum, an undergraduate degree and an annual household income greater than CAD 70,000 (Table 1). Almost all mothers were married, a nearly equal number of children were assigned male and female sexes at birth, and almost all children were born at greater than 37 weeks’ gestation (Table 1).

## 6. Exploratory Phase: Determining Relevant Variables

Using an exploratory approach, a bivariate correlation matrix was conducted to determine all relevant predictor and mediator variables that would be included in the models (Supplementary Materials, Table S1).

### 6.1. Identifying Statistically Relevant Predictor Variables

Bivariate correlation analysis revealed that no prenatal variables were significantly correlated with children’s insecure versus secure attachment patterns (Supplementary Materials, Table S1). Therefore, no further hypothesis testing was conducted for predictors of insecure versus secure attachment patterns. Regarding disorganized versus organized attachment patterns, mothers’ prenatal cortisol (AUCg) was correlated with disorganized versus organized attachment patterns (Supplementary Materials, Table S1); therefore, mothers’ prenatal cortisol (AUCg) was examined as a predictor of children’s disorganized versus organized attachment patterns. Mothers’ prenatal cortisol (AUCi) was not correlated with disorganized versus organized attachment and was therefore dropped from further analysis (Supplementary Materials, Table S1). Though mothers’ depressive symptoms were not correlated with either type of attachment, early-pregnancy depressive symptoms were included as a covariate in the adjusted models due to observed associations with child development [8,9,59,60,61]. Further, prenatal social support was not included as a moderator as prenatal depressive symptoms were eliminated from the model (see Figure 1). However, social support at the time of maternal cortisol collection was concontrolled, as theoretically important.

### 6.2. Identifying Statistically Relevant Mediators

In identifying relevant postpartum mediators, infants’ compulsive and passive behaviours in mother–child interactions were found to correlate with disorganized versus organized attachment patterns (Supplementary Materials, Table S1). Further, infants’ cortisol at 6 months (AUCi) was correlated with their mothers’ prenatal cortisol (AUCg) and with children’s compulsive behaviours in parent–child interactions, while infants’ passive behaviours in parent–child interactions were correlated with their mothers’ prenatal cortisol (Supplementary Materials, Table S1). Therefore, analyses were used to explore infants’ cortisol at 6 months (AUCi) and infants’ compulsive and passive tendencies in parent–child interactions as postpartum mediators of the association between mothers’ prenatal cortisol (AUCg) and their children’s disorganized versus organized attachment patterns. Given theoretical understandings and observed correlations, analyses considered indirect effects and sequential mediation of mothers’ prenatal cortisol (AUCg), infants’ cortisol (AUCi), and infants’ compulsive or passive tendencies in parent–child interactions. Postnatal social support was not included as a moderator as postnatal depressive symptoms were eliminated from the model (see Figure 1).

## 7. Models Including Adjustment for Relevant Covariates

### 7.1. Direct Effects of Mothers’ Prenatal Cortisol on Children’s Disorganized Attachment

**Figure 2:**
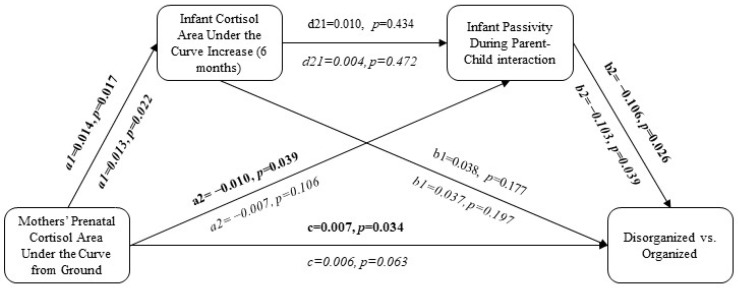
Predicting disorganized versus organized attachment using maternal cortisol given infant cortisol and passivity during parent–infant interactions. The adjusted model (covariates = maternal ethnicity and income, children’ birthweight, early-pregnancy depressive symptoms, and early-pregnancy social support) is represented in italics. Bolded pathways represent statistically significant pathways.

**Figure 3:**
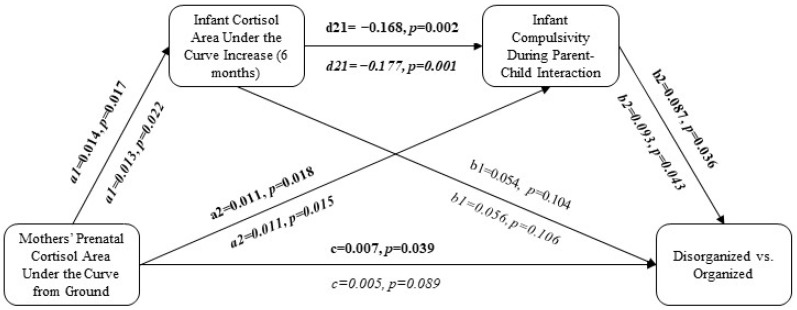
Predicting disorganized versus organized attachment pattern using maternal cortisol given infant cortisol and compulsivity during parent–child interactions. The adjusted model (covariates = maternal ethnicity and income, children’ birthweight, early-pregnancy depressive symptoms, and early-pregnancy social support) is represented in italics. Bolded pathways represent statistically significant pathways.

Mothers’ prenatal cortisol (AUCg) significantly predicted disorganized versus organized attachment (*p* < 0.04; Figure 2 and Figure 3). After adjustment for relevant covariates, mothers’ prenatal cortisol (AUCg) no longer statistically significantly predicted disorganized versus organized attachment (*p* > 0.05; Figure 2 and Figure 3).

### 7.2. Effects of Postnatal Variables on Children’s Disorganized Attachment

Mothers’ prenatal cortisol (AUCg) significantly predicted infant cortisol (AUCi) in the unadjusted (*p* < 0.02) and adjusted (*p* < 0.03) analyses, but infant cortisol (AUCi) did not significantly predict disorganized versus organized attachment patterns in either the unadjusted or adjusted analyses (*p* > 0.05; Figure 2 and Figure 3). In the unadjusted analyses, mothers’ prenatal cortisol (AUCg) significantly predicted both passivity (*p* < 0.04) and compulsivity (*p* < 0.02) among the infants during parent–child interactions, and passivity (*p* < 0.03) and compulsivity (*p* < 0.04) predicted disorganized versus organized attachment (Figure 2 and Figure 3). Following adjustment for relevant covariates, mothers’ prenatal cortisol (AUCg) no longer predicted children’s passivity in parent–child interactions (*p* > 0.10; Figure 2), but it continued to predict children’s compulsivity (*p* < 0.02), which, in turn, predicted disorganized versus organized attachment (*p* < 0.05; Figure 3).

### 7.3. Serial Mediation Pathways

When combined in serial mediation: (1) mothers’ prenatal cortisol (AUCg) predicted infant cortisol (AUCi), (2) infant cortisol (AUCi) predicted infant compulsivity (but not passivity) during parent–child interactions, and (3) infant compulsivity predicted disorganized versus organized attachment (Figure 2 and Figure 3). However, all 95% bootstrap intervals were insignificant, suggesting that the indirect effects were not statistically significant (Table 2 and Table 3).

## 8. Models Including the Residualized Mothers’ Prenatal Cortisol Predictor Variable

### 8.1. Direct Effects of Mothers’ Prenatal Cortisol on Children’s Disorganized Attachment

**Figure 4:**
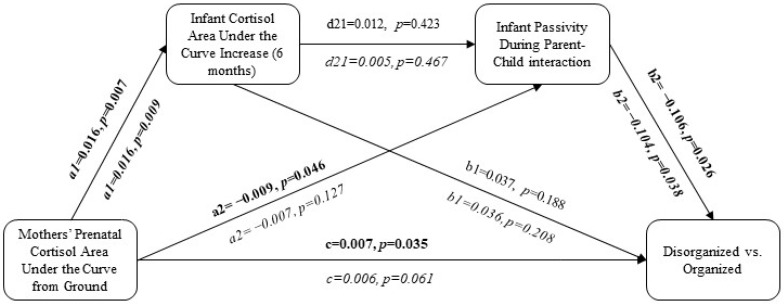
Predicting disorganized versus organized attachment given infant passivity in parent–infant interactions. Mothers’ prenatal cortisol was a residualized value derived from the linear regression of mothers’ prenatal cortisol and gestational age at the time of cortisol data collection. The adjusted model (covariates = maternal ethnicity and income, children’ birthweight, early-pregnancy depressive symptoms, and early-pregnancy social support) is represented in italics. Bolded pathways represent statistically significant pathways.

**Figure 5:**
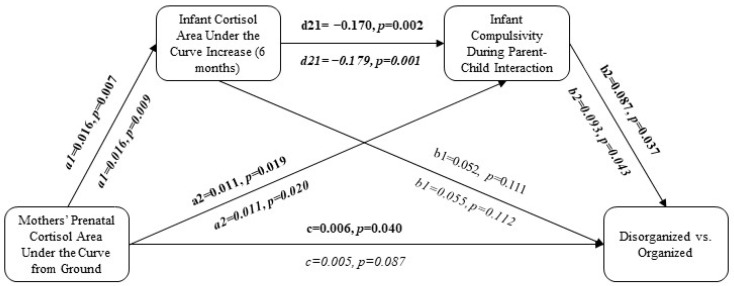
Predicting disorganized versus organized attachment patterns given infant compulsivity in parent–child interactions. Mothers’ prenatal cortisol was a residualized value derived from the linear regression of mothers’ prenatal cortisol and gestational age at the time of cortisol data collection. The adjusted model (covariates = maternal ethnicity and income, children’ birthweight, early-pregnancy depressive symptoms, and early-pregnancy social support) is represented in italics. Bolded pathways represent statistically significant pathways.

After including residuals from the linear regression between mothers’ prenatal cortisol (AUCg) and gestational age into the model, mothers’ prenatal cortisol continued to directly predict children’s disorganized versus organized attachment patterns in the unadjusted models (*p* < 0.05; Figure 4 and Figure 5).

### 8.2. Effects of the Postnatal Variables on Children’s Disorganized Attachment

After including the residuals from the linear regression of mothers’ prenatal cortisol (AUCg) against gestational age, mothers’ prenatal cortisol (AUCg) continued to predict infants’ cortisol (AUCi) at 6 months (*p* < 0.01); however, infant cortisol (AUCi) did not predict disorganized versus organized attachment in either the adjusted or unadjusted analyses (*p* > 0.05; Figure 4 and Figure 5). Mothers’ prenatal cortisol (AUCg) predicted infant passivity in parent–child interactions only in the unadjusted analysis (*p* < 0.05; Figure 4), but it predicted infant compulsivity in both the unadjusted and adjusted analyses (*p* < 0.03; Figure 5). Infant passivity only predicted disorganized versus organized attachment in the unadjusted analysis (*p* < 0.03; Figure 4), while infant compulsivity in parent–child interactions continued to predict disorganized versus organized attachment in both the unadjusted and adjusted analyses (*p* < 0.05; Figure 5).

### 8.3. Serial Mediation Pathways

When combined in serial mediation: (1) mothers’ prenatal cortisol (AUCg) predicted infant cortisol (AUCi), (2) infant cortisol (AUCi) predicted infant compulsivity (but not infant passivity) during parent–child interactions, and (3) infant compulsivity predicted disorganized versus organized attachment in both the unadjusted and adjusted models (Figure 4 and Figure 5). However, all 95% bootstrap intervals were insignificant, suggesting that the indirect effects were not statistically significant (Table 4 and Table 5).

## 9. Discussion

Children’s insecure and disorganized attachment patterns may be predicted by variables that influence the quality of their interactions with their mothers, including prenatal and postnatal exposures and their own behaviours. In this exploratory study we aimed to identify: (1) prenatal predictors of infants’ attachment patterns and (2) postnatal mediators of the associations between predictors and children’s attachment patterns, adjusted for relevant sociodemographic factors. No variables appeared to correlate with children’s insecure versus secure attachment patterns; therefore, insecure versus secure attachment was not modeled. Our hypothesis that more depressive symptoms and greater prenatal cortisol levels in mothers would predict disorganized (versus organized) attachment was partially supported, with only mothers’ prenatal cortisol (AUCg) predicting disorganized attachment (versus organized attachment). More specifically, mothers’ prenatal cortisol (AUCg) appeared to directly predict children’s disorganized (versus organized) attachment without adjustment. Though no significant indirect effects were observed for disorganized attachment, mothers’ prenatal cortisol (AUCg) did predict: (1) infants’ compulsivity (unadjusted and adjusted) and passivity (unadjusted) during parent–child interactions, which predicted disorganized versus organized attachment in all models, and (2) infants’ cortisol (AUCi) at 6 months, which then predicted infant compulsivity, and this, in turn, predicted disorganized versus organized attachment in all models. Therefore, findings from this study suggest that maternal and child cortisol levels may predict disorganized versus non-disorganized attachment in children. Further, the results allude to the influential role children play in parent–child interactions and that their behaviors during such interactions may be predicted by mothers’ prenatal cortisol and could contribute to the development of disorganized versus organized attachment patterns.

### 9.1. Mothers’ Prenatal Cortisol Levels and Postnatal Mediators and Outcomes

While there is some debate on the applicability, this study employed salivary cortisol as a proxy for chronic stress in mothers, as HPA axis activity and subsequent cortisol output is affected by chronic stress [102]. Evidence suggests that four days of salivary cortisol measurements may be necessary to characterize stable trait-like AUC stress responses in humans [103], though some suggest that two days are moderately reliable for measuring specific AUC responses such as AUC total [104,105]. We aggregated two days of diurnal salivary cortisol measurements taken during early gestation to produce a more stable estimate of chronic stress. Diurnal salivary cortisol patterns also correlate with adverse social stressors that are implicated in causing chronic stress [102,106], and are used to measure changes across various physiological systems (e.g., the cardiovascular system) in addition to the HPA axis [107]. The non-invasive and rapid nature of salivary cortisol collection has been referred to as an accessible, objective measure of chronic stress, particularly when collected across multiple days [107]. Lastly, mothers’ salivary cortisol levels have been associated with their perceived levels of stress, burnout symptoms, chronic pain levels, and being divorced or widowed [108], which reflect chronic stress and reinforce using salivary cortisol as a proxy.

Mothers’ prenatal cortisol directly predicted children’s disorganized versus organized attachment patterns in all models. It also predicted infant cortisol levels in all models, supporting the existing literature on the role that mothers’ prenatal stress plays in infants’ postnatal cortisol production [109,110,111]. This finding supports the fetal programming hypothesis, which suggests that children’s HPA functioning can be programmed through gene-by-environment interactions as early as the prenatal period [50,69]. While Attachment Theory posits that one’s attachment pattern is mainly determined by social factors and, in particular, the quality of primary caregiver/parent sensitivity and responsiveness in interactions [112], the findings from this study suggest that maternal cortisol levels during pregnancy should be further examined for their role in the development of disorganized versus organized attachment patterns.

In alignment with the fetal programming hypothesis [48], mothers who experience heightened levels of cortisol during pregnancy likely transmit more cortisol to their developing fetus(es) through inactivation of the enzyme that is responsible for governing cortisol levels during pregnancy vis-à-vis stress exposure (i.e., 11beta-hydroxysteroid dehydrogenase type 2) [50]. Consequently, elevated cortisol exposure might signal the developing fetus to prepare for a stressful environment postnatally, altering fetal HPA development to match expectations [50]. While likely adaptive under some circumstances, an overactive HPA system can challenge infants’ emotional regulation, executive functioning, and fear response [113], potentially undermining their capacity to optimally interact with their parents [21]. Further, mothers’ prenatal cortisol levels predicted children’s passivity and compulsivity during parent–child interactions at 6 months of age, revealing the importance of prenatal cortisol exposure to children’s interactive behaviours and a potential precursor to the development of the disorganized attachment pattern. Infants’ cortisol levels at 6 months were also associated with their compulsivity in interactions, suggesting intergenerational impacts of exposure to altered HPA functions on concurrent alterations and associated challenging behaviour in mother-child interactions.

While the disorganized attachment pattern is theorized to develop when infants are exposed to extremely atypical caregiving behaviours [28,29], other factors have also been investigated to explain the pattern [28,29,30]. Our findings and others’ have suggested that disorganized attachment may also be affected mechanistically by alterations in HPA functioning [114]. A flatter diurnal cortisol slope has been observed among infants with disorganized attachment compared to non-disorganized infants [115], and adolescents who demonstrated disorganized attachment patterns as infants, demonstrated hyperresponsive HPA function [114]. A systematic review revealed larger amygdala volumes or activation among infants with disorganized attachment [116] and prenatal cortisol exposure has been linked to both larger infant amygdala volume and a more active HPA axis [117]. Therefore, exposure to prenatal cortisol could possibly increase the risk of developing disorganized attachment through its indirect effects involving brain structure and HPA functioning [116]. Further study of the links among prenatal and postnatal cortisol, children’s brain morphology, and children’s disorganized attachment patterns are needed to further explore these putative mechanisms.

### 9.2. Children Are Influential Partners during Parent–Child Interactions

Child passivity (in unadjusted models) and compulsivity (in adjusted and unadjusted models) during parent–child interactions significantly predicted disorganized versus organized attachment patterns. These novel findings affirm the instrumental role that children play during parent–child interactions, but they also suggest that children may be primed via exposure to high cortisol during gestation to interact in ways that challenge parents in interactions via being passive or compulsive [118]. Previous research has demonstrated that children exposed to high prenatal cortisol demonstrate more crying, fussing, and negative facial expressions that can challenge parents during interactions [119]; however, to our knowledge, researchers have not observed passivity or compulsivity associated with early prenatal cortisol exposure. This important finding alludes to the importance of intervening during pregnancy (e.g., reducing mothers’ stress) to prevent infants from developing challenging behaviours that contribute to suboptimal parent–child interactions and, ultimately, undermine the development of secure attachment.

### 9.3. Secure Versus Insecure Attachment: Null Findings

Consistent with the other findings, this study did not identify variables that sufficiently explained the variance in secure attachment pattern development [6,7,8,9]. Many studies that examine predictors of child attachment security do not consider, or scarcely touch on, the child’s role in establishing the quality of the bidirectional connection between mothers and their infants [120]. Though studies have identified relatively robust evidence for predictors of children’s insecure attachment patterns (e.g., avoidant, ambivalent/resistant), these studies largely focus on the parents’ role in child attachment development. Considering the role of the child in shaping interactions with their parents may be specifically useful for improving the understanding of secure attachment. The null findings could also be attributable to the high socioeconomic status of this sample [8,64,65,66,67], which may have served as a protective factor that promotes parent–child interaction quality [121]. Further, children who reside in what appear to be safe, sensitive, and responsive caregiving environments can still develop insecure attachment patterns [122], suggesting that children’s behaviours in interactions, or other inherent characteristics (e.g., genetics, temperament), may play prominent roles in attachment pattern development. This possibility requires further study.

### 9.4. Impacts of Relevant Covariates

Some of the findings in this paper were no longer statistically significant after controlling for statistically relevant (i.e., maternal ethnicity and income and child birthweight) and theoretically relevant (i.e., early pregnancy maternal depressive symptoms and social support) covariates. Since these sociodemographic factors are closely linked with the caregiving environment, children’s health, and parent–child interaction quality [8,66,67,69,70], the confounding influence on the outcome of interest was unsurprising. However, after adjustments, this exploratory study continued to reveal statistical significance in the sequential mediation pathway from mothers’ prenatal cortisol to children’s disorganized attachment through infants’ cortisol and their compulsive behaviours in parent–child interactions. This may underscore the particular importance of children’s compulsive behaviours in parent–child interactions (alongside mothers’ and infants’ cortisol levels) in the genesis of disorganized attachment. This finding is biologically plausible considering that exposure to high prenatal cortisol may induce infant HPA alterations that affect both infants’ interactive behaviours and attachment patterns, especially disorganization [114]. However, this requires further testing and replication, ideally with higher risk, clinical samples.

### 9.5. Implications for Intervention

Intervening to address factors associated with mothers’ elevated cortisol levels during early pregnancy may be an important primordial or primary preventive effort to reduce intergenerational transmission of HPA axis hyperactivity and associated health sequelae (e.g., poor social interactions and disorganized attachment) [50,113]. Higher levels of cortisol among women considering pregnancy are linked to financial factors, inadequate social support (particularly from intimate/marital partners), personal experiences of childhood adversity, and mental health concerns, especially anxiety [123]. Primary prevention that addresses these factors prior to pregnancy could promote conditions conducive to less stressful pregnancy and subsequently prevent the development of disorganized attachment in children. For example, Hidayati et al. reported on an interactive pregnancy education (IPE) intervention that included education on the adverse effects of stress through a counseling-based platform, breathwork practice, and yoga; this intervention reduced cortisol and stress levels among pregnant women, particularly those who were highly stressed [124]. Further, the current study’s findings about 6-month-old infants’ behaviours in mother-infant interactions suggests that behavioural precursors to attachment patterns may be observed much earlier than the expected 9 months of age. Passivity and compulsivity in interactions could be considered early warning signs of the development of disorganized attachment pattern. In addition to early developmental screening and assessments that can prevent related behavioural problems in at-risk young children [125], postpartum interventions such as Attachment and Child Health (ATTACH^TM^) can promote secure attachment through targeting improvement in parents’ reflective functioning that provides them with better insight into their children’s thoughts, feelings, and mental states [125,126].

### 9.6. Limitations, Strengths, and Future Directions

The generalizability of this study may be limited by the high socioeconomic, non-clinical sample; however, the findings may still be applicable to populations of highly educated, high-income, and low-risk white mothers and their children. Despite null findings for identifying predictors of secure versus insecure attachment, the findings are supported by the literature, demonstrating that predictors of secure attachment are more difficult to pinpoint than for disorganized attachment [6,8,9]. This study may also be limited by the inclusion of categorical rather than continuous measures of attachment. This approach considers all children in one category to be of similar severity and may also increase the risk of misclassification. However, this study employed the most-reliable gold standard measure of child attachment [127], and the data were coded by a certified reliable coder who attained inter-rater reliability with a highly skilled SSP trainer, validating this coding. Nonetheless, future studies could consider utilizing concomitant measures of categorical and continuous measures of attachment patterns. Further, salivary cortisol levels may be limited as a proxy for maternal chronic stress on child development; however, they correlate with maternal blood cortisol levels and higher levels of perceived stress [108]. This study team sought to produce a more stable estimate of cortisol by aggregating two days of diurnal salivary cortisol measurements that were taken during early gestation. The variation in data collection for the mothers’ cortisol due to differences and challenges in scheduling and associated family dynamics (e.g., employment and childcare) may have also limited the findings. Future studies could consider hair or nail samples for cortisol, as proxies for chronic stress that are less prone to variation [108]. Finally, breastfeeding has also been identified as a potential contributor to children’s attachment [128] and socio-emotional development [129]; however, as the study’s focus was on psychosocial and physiological predictors of attachment patterns, breastfeeding was not considered. Future research could consider the role of breastfeeding in the context of children’s attachment patterns. Overall, findings for disorganized attachment are supported by the literature and embody plausible biological mechanisms. This may be the first study to report on the associations among prenatal HPA function (cortisol), children’s behaviours in interactions with parents, and disorganized attachment.

## 10. Conclusions

Findings from this exploratory study suggest that maternal cortisol levels during pregnancy, infant cortisol, and children’s observable behaviours during parent–child interactions might predict children’s disorganized (versus organized) attachment patterns through various pathways. Intergenerational impacts of prenatal stress, as assessed by maternal cortisol, may negatively affect infants’ capacity to engage with their primary caregivers/parents, predisposing them to developing disorganized attachment patterns. Clinicians could be advised to assess parent–child interaction relationship quality, with a focus on children’s behaviours in earlier interactions, to identify children at risk of developing disorganized attachment. This knowledge may support health professionals in preventing a cascade of adverse health outcomes that can arise from disorganized attachment patterns.

## Figures and Tables

**Table 1:** Participant demographic information (*n* = 214).

Variables	Frequency or Mean (SD)	Percentage (%)
**Sociodemographic Variables**		
*Maternal Education*		
Below university degree	60	28
University degree or more Missing data	151 3	70.6 1.4
*Ethnicity*		
White	172	80.4
Non-White Missing data	40 2	18.7 0.9
*Marital Status*		
Single	2	0.9
Married Missing data	209 3	97.6 1.4
*Parity*		
0	107	50.0
1	82	38.3
2 or more Missing data	24 1	11.2 0.5
*Household Income*		
Less than CAD 70 k	39	18.2
CAD 70 k or more Missing data	169 6	79.0 2.8
*Gestational Age at Birth*		
<37 weeks	10	4.7
37 and more weeks Missing data	202 2	94.4 0.9
*Child Sex*		
Male	108	50.4
Female Missing data	105 1	49.1 0.5
*Mothers’ Age (years)*	31.8 (3.84)	
*Birthweight (grams)*	3365 (531)	
*Child Age at Attachment Assessment (months)*	22.1 (4.43)	
**Predictors and Mediators**		
*Parent–Child Interaction* Infant passivity	4.55 (3.71)	
Infant compulsivity	1.89 (3.64)	
Infant difficulty	2.77 (2.74)	
*Cortisol* Maternal cortisol (area under the curve ground) at mean 15 (SD = 3.7) weeks’ gestation (μg/dL) Maternal cortisol (area under the curve increase) at mean 15 (SD = 3.7) weeks’ gestation (μg/dL) Child cortisol (area under the curve ground) at 6 months (μg/dL) Child cortisol (area under the curve increase) at 6 months (μg/dL)	1.73 (0.47) 2.42 (3.29) 0.13 (0.07) 0.004 (0.04)	
**Outcomes**		
*Disorganized vs. Organized Attachment (Secure or Insecure)*		
Disorganized	35	16.4
Organized	179	83.6
*Secure vs. Non-Secure Attachment (Insecure or Disorganized)*		
Secure	99	46.3
Non-secure	115	53.7

**Table 2:** Indirect effects of maternal pregnancy cortisol through infant passivity on child attachment (disorganized versus organized).

Indirect Effect	Effect	Bootstrap SE	95% CI
Total Indirect Effect	*0.0015*	*0.0012*	*−0.0003*, *0.0045*
	*0.0012*	0.0012	−0.0006, 0.0042
Ind 1 (a): maternal prenatal cortisol (AUCg) → infant cortisol (AUCi) → disorganized vs. organized	*0.0005*	*0.0008*	*−0.0005*, *0.0026*
	*0.0005*	0.0009	−0.0006, 0.0030
Ind 2 (b): maternal prenatal cortisol (AUCg) → infant passivity → disorganized vs. organized	*0.0010*	*0.0008*	*−0.0003*, *0.0029*
	*0.0007*	0.0008	−0.0006, 0.0025
Ind 3 (c1): maternal prenatal cortisol (AUCg) → infant cortisol (AUCi) → infant passivity → disorganized vs. organized	*0.0000*	*0.0001*	*−0.0002*, *0.0002*
	*0.0000*	0.0001	−0.0002, 0.0003

Note: The adjusted model (covariates = maternal ethnicity and income, children’ birthweight, early-pregnancy depressive symptoms, and early-pregnancy social support) is represented in italics; effect = standardized regression, SE = standard error, and CI = confidence interval.

**Table 3:** Indirect effects of maternal pregnancy cortisol through infant compulsivity on child attachment (disorganized versus organized).

Indirect Effect	Effect	Bootstrap SE	95% CI
Total Indirect Effect	*0.0015*	*0.0012*	*−0.0006*, *0.0043*
	*0.0016*	0.0014	−0.0007, 0.0048
Ind 1 (a): maternal prenatal cortisol (AUCg) → infant cortisol (AUCi) → disorganized vs. organized	*0.0007*	*0.0010*	*−0.0004*, *0.0032*
	*0.0008*	0.0011	−0.0005, 0.0037
Ind 2 (b): maternal prenatal cortisol (AUCg) → infant compulsivity → disorganized vs. organized	*0.0010*	*0.0008*	*−0.0006*, *0.0027*
	*0.0011*	0.0010	−0.0008, 0.0033
Ind 3 (c1): maternal prenatal cortisol (AUCg) → infant cortisol (AUCi) → infant compulsivity → disorganized vs. organized	*−0.0002*	*0.0002*	*−0.0008*, *0.0001*
	*−0.0002*	0.0003	−0.0009, 0.0001

Note: The adjusted model (covariates = maternal ethnicity and income, children’ birthweight, early-pregnancy depressive symptoms, and early-pregnancy social support) is represented in italics; effect = standardized regression, SE = standard error, and CI = confidence interval.

**Table 4:** Indirect effects of mothers’ prenatal cortisol residualized on gestational age at the time of cortisol data collection through infant passivity on child attachment (disorganized versus organized).

Indirect Effect	Effect	Bootstrap SE	95% CI
Total Indirect Effect	*0.0016*	*0.0013*	*−0.0004*, *0.0046*
	*0.0012*	0.0013	−0.0008, 0.0044
Ind 1 (a): maternal prenatal cortisol (AUCg) → infant cortisol (AUCi) → disorganized vs. organized	*0.0006*	*0.0009*	*−0.0006*, *0.0029*
	*0.0006*	0.0010	−0.0008, 0.0033
Ind 2 (b): maternal prenatal cortisol (AUCg) → infant passivity → disorganized vs. organized	*0.0010*	*0.0008*	*−0.0003*, *0.0029*
	*0.0007*	0.0008	−0.0007, 0.0024
Ind 3 (c1): maternal prenatal cortisol (AUCg) → infant cortisol (AUCi) → infant passivity → disorganized vs. organized	*0.0000*	*0.0001*	*−0.0003*, *0.0003*
	*0.0000*	0.0001	−0.0003, 0.0003

Note: The adjusted model (covariates = maternal ethnicity and income, children’ birthweight, early-pregnancy depressive symptoms, and early-pregnancy social support) is represented in italics; effect = standardized regression, SE = standard error, and CI = confidence interval.

**Table 5:** Indirect effects of mothers’ prenatal cortisol residualized on gestational age at the time of cortisol data collection through infant compulsivity on child attachment (disorganized versus organized).

Indirect Effect	Effect	Bootstrap SE	95% CI
Total Indirect Effect	*0.0015*	*0.0013*	*−0.0007*, *0.0044*
	*0.0016*	0.0015	−0.0008, 0.0050
Ind 1(a): maternal prenatal cortisol (AUCg) → infant cortisol (AUCi) → disorganized vs. organized	*0.0008*	*0.0011*	*−0.0005*, *0.0036*
	*0.0009*	0.0012	−0.0005, 0.0041
Ind 2 (b): maternal prenatal cortisol (AUCg) → infant compulsivity → disorganized vs. organized	*0.0010*	*0.0008*	*−0.0007*, *0.0027*
	*0.0010*	0.0010	−0.0009, 0.0033
Ind 3 (c1): maternal prenatal cortisol (AUCg) → infant cortisol (AUCi) → infant compulsivity → disorganized vs. organized	*−0.0002*	*0.0003*	*−0.0009*, *0.0001*
	*−0.0003*	0.0003	−0.0010, 0.0001

Note: The adjusted model (covariates = maternal ethnicity and income, children’ birthweight, early-pregnancy depressive symptoms, and early-pregnancy social support) is represented in italics; effect = standardized regression, SE = standard error, and CI = confidence interval.

## Data Availability

The data associated with this study have not been deposited into a publicly available repository; however, the data will be made available on request to the corresponding author.

## Supplementary Materials

The following supporting information can be downloaded at: https://www.mdpi.com/article/10.3390/children11081022/s1, Table S1: Bivariate correlational matrix (*n* = 214); Table S2: Exploratory findings from univariate linear regression models.
