# Supplementary material for: Perinatal Predictors and Mediators of Attachment Patterns in Preschool Children: Exploration of Children’s Contributions in Interactions with Mothers
Source: Children (Basel). 2024 Aug 21;11(8):1022. doi: 10.3390/children11081022 (PMC11352242; doi:10.3390/children11081022)
Supplement: Supplementary file 1 [file children-11-01022-s001.zip › children-3113921-supplementary.pdf]

Supplementary Materials

Table S2. Exploratory findings from univariate linear regression models.

Variable	Secure vs. Non-Secure P-value	Disorganized vs. Organized P-value
Maternal Education (ref: Below university degree)		
University degree or more	0.436	0.388
Ethnicity (ref: Caucasian)		
Non-Caucasian	0.942	*0.022**
Marital Status (ref: Single)		
Married	0.647	0.389
Parity	0.591	0.893
Household Income (ref: Less than 70k)		
70k or more	0.57	*0.086**
Gestational Age at Birth		
37 and more weeks (ref:< 37 weeks)	*0.074**	0.563
Child Sex (ref: male);		
Female	0.307	0.316
Maternal Age	0.317	0.72
Birthweight	*0.176**	*0.22**
Child age at attachment assessment	0.743	0.68

Note: A p-value of 0.25 or lower was deemed moderately significant.

Table S1. Bivariate correlational matrix**.**

	**1**	**2**	**3**	**4**	**5**	**6**	**7**	**8**	**9**	**10**	**11**	**12**	**13**	**14**	**15**	**16**	**17**
Disorganized versus Non-Disorganized [1]																	
Secure Vs. Non-Secure [2]	**-0.41***																
CortAUCg_ADJ [3]	**0.17***	-0.01															
CARTadj [4]	-0.03	-0.03	**0.26 ***														
CART3adj [5]	-0.07	0.09	0.01	0.11													
CortAUCg_T3ADJ [6]	0.09	-0.04	**0.45***	0.07	0.09												
Social Support Early Pregnancy [7]	0.06	-0.03	0.04	-0.02	0.08	0.05											
Social Support Late Pregnancy [8]	0.09	0.01	0.08	0.09	0.04	0.05	**0.73***										
Social Support 3 Months Postpartum [9]	0.07	-0.02	-0.03	-0.06	0.07	-0.03	**0.56***	**0.67***									
Maternal Depression Early Pregnancy [10]	-0.04	0.06	0.02	0.05	-0.11	-0.09	**-0.37***	**-0.34***	**-0.24***								
Maternal Depression Late Pregnancy [11]	-0.09	0.07	-0.02	0.00	-0.06	-0.04	**-0.33***	**-0.38***	**-0.33***	**0.64***							
Maternal Depression 3 Months Postpartum [12]	-0.06	0.04	0.10	0.08	-0.02	0.09	**-0.23***	**-0.21***	**-0.33***	**0.49***	**0.47***						
Cooperative [13]	0.03	-0.04	0.07	0.10	-0.01	0.01	0.02	-0.02	-0.02	-0.06	-0.05	0.04					
Difficult [14]	0.02	-0.03	-0.04	0.00	0.05	-0.04	0.01	0.08	0.12	0.07	0.04	-0.01	-0.02				
Compulsive [15]	**0.13+**	0.01	0.11	-0.07	-0.08	0.01	-0.10	-0.12	-0.03	0.12+	0.05	-0.01	**-0.35***	**-0.28***			
Passive [16]	**0.15**	0.03	***-0.11***	0.02	0.04	0.01	0.07	0.06	-0.06	***-0.14***	-0.04	-0.00	**-0.18***	**-0.49***	**-0.58***		
Infant Cortisol AUCg [17]	-0.02	0.02	**-0.12+**	-0.03	0.04	-0.10	-0.01	0.07	0.05	-0.05	-0.02	0.06	0.01	0.03	-0.06	0.02	
Infant Cortisol AUCi [18]	0.09	0.04	**0.17***	0.07	**0.16 ***	0.04	0.03	0.03	0.015	-0.05	-0.04	-0.04	0.03	**0.23***	**-0.18***	-0.01	**0.27**

Notes:
*p-value< 0.05
+ p-value<0.10
AUCg = Area Under the Curve from Ground
AUCi = Area Under the Curve Increase

CortAUCg_ADJ = Maternal Prenatal Cortisol AUCg Early pregnancy
CortAUCg_T3ADJ [6] = Maternal Prenatal Cortisol AUCg late pregnancy
CARTadj [4] = Maternal Prenatal Cortisol AUCi early pregnancy
CART3adj [5] =Maternal Prenatal Cortisol AUCi late pregnancy
